# Introductory lecture: Biochemical properties of food allergens

**DOI:** 10.1186/2045-7022-1-S1-S34

**Published:** 2011-08-12

**Authors:** Lars K Poulsen

**Affiliations:** 1 National University Hospital, Allergy Clinic, Copenhagen, Denmark

## 

The biochemical properties of food allergens exert a large influence on how these allergens act when ingested, and determines important aspects such as cross-reactivity, lability to degradation during digestion or stability during processing of the food. One of the targets for biochemical characterization of food allergens is to establish a link between the biochemical data and the biological activity of the allergen in a certain food. The biological activity of food allergens or mixtures thereof may be determined by various in vivo and in vitro methods that may quantitatively or semi-quantitatively express the combined effects of individual allergenic molecules in a mixture. Even in the rare case of testing an individual food allergen molecule, a response will emerge that is only declared relatively to other allergenic substances or mixtures. Thus, an important feature of testing the biological activity of mixtures is the lack a response which can be directly linked to individual molecular entities, and this put special emphasis on the definition of both the test systems and the mixtures that are tested. As an example, the lessons learned by the recent outbreak of reactions to the socalled Meripro 711, a wheat-derived hydrolyzed protein, will be discussed in the context of what is known about the type of patients that react, and how this may be related to the biochemical properties of the product.

